# Bonding Characteristics of Silane Coupling Agent and MMA-Containing Primer to Various Composite CAD/CAM Blocks

**DOI:** 10.3390/polym15163396

**Published:** 2023-08-13

**Authors:** Masaki Asakura, Koki Aimu, Tatsuhide Hayashi, Masakazu Matsubara, Akimichi Mieki, Seiji Ban, Tatsushi Kawai

**Affiliations:** Department of Dental Materials Science, School of Dentistry, Aichi Gakuin University, 1-00 Kusumoto-cho, Nagoya 464-8650, Japan; masaki@dpc.agu.ac.jp (M.A.); skoidats@gmail.com (K.A.); masakazu@dpc.agu.ac.jp (M.M.); akimichi.mieki@gmail.com (A.M.); sban@g.agu.ac.jp (S.B.); kwaita@dpc.agu.ac.jp (T.K.)

**Keywords:** bond strength, composites, CAD/CAM, sandblast, resin primer, silane coupling agent

## Abstract

This study evaluated the bonding characteristics of a silane coupling agent (SCA) and a methyl methacrylate (MMA)-containing primer (MCP) for 11 types of commercial composite blocks (CBs) for sandblasted and non-sandblasted surfaces. The shear bond strength (SBS) was measured according to ISO 29022: Notched-edge shear bond strength test. The SBS results demonstrated statistically significant differences between the CBs under all identical conditions. For the non-sandblasted groups, the SBSs of MCP-treated specimens were significantly higher than those of SCA-treated specimens for all but two CBs. Comparing the two treatments in sandblasted groups, the SBS was significantly higher for seven out of 11 MCP-treated RCB specimens, in contrast with three cases for the SCA-treated group. Two-way ANOVA for SBS showed the interaction effect between sandblasting and primer type for specific CBs, indicating that the sandblasting treatment improved SBS more effectively for SCA-treated specimens. Moreover, the effect of the SCA treatment was more material-dependent compared to that of the MCP treatment, which did not achieve a strong bond in all CBs but proved more effective than the SCA treatment, especially for non-sandblasted surfaces.

## 1. Introduction

In recent years, the demand for metal-free and esthetic restorations has increased. With the development of computer-aided design/computer-aided manufacturing (CAD/CAM), the use of ceramics such as zirconia and composite blocks (CBs) for CAD/CAM has increased in clinical practice [1]. CBs represent a diverse array of materials with a growing spectrum of properties, falling into two distinct categories based on their microstructure: dispersed filler and polymer-infiltrated ceramic network (PICN) materials [2]. PICNs differ notably from traditional composite materials with dispersed fillers, where the fillers are mixed into a matrix. Instead, PICNs are crafted by infiltrating pre-sintered glass-ceramic scaffolds with a monomer [2], enabling more efficient stress distribution in all directions and enhancing resistance to breakdown phenomena [3]. While ceramics have superior mechanical strengths and esthetic properties, CBs have advantages such as reduced antagonist enamel wear [4], an elastic modulus closer to that of dentin allowing them to absorb masticatory force [5], and better suitability for milling thin-shaped margins [6]. Consequently, CBs have attracted attention as materials for single crowns on natural teeth and implants, and for bonded partial restorations such as inlays and onlays [2].

However, their applicability is hampered by the fact that it is difficult to achieve an adequate bonding with resin cements, with several studies reporting on clinical outcomes related to crown debonding [7,8,9,10]. The most recent study revealed that 362 CAD/CAM composite crowns were assessed over an average follow-up period of 378 d. Among these, 106 crowns exhibited clinical issues, with crown debonding accounting for 74.5% of the cases. Notably, a relatively higher rate of debonding was observed in the post-fitting period. Furthermore, after the reattachment of the debonded crowns, only 16% of them (12 crowns) experienced subsequent debonding, while the rest remained intact for an extended period [7]. A similar pattern was reported by Kabetani et al., who found that 50% of debondings occurred within the first 4 months after cementation of CAD/CAM composite crowns. Additionally, the authors noted that alumina-blasting and silane treatment were carried out in all cases of debonding [8].

CBs are produced by curing under high pressure and temperature in factories. This process leads to good mechanical properties because of the high degree of conversion [11], but makes it difficult to achieve chemical bonding between the resin matrix of CBs and resin cements owing to the lack of free monomers [2,12]. Therefore, a silane coupling agent (SCA) is commonly used as a primer to achieve chemical bonding with the fillers in CBs [13,14]. In recent years, an alternative approach for bonding has been introduced, which uses a methyl methacrylate (MMA)-containing primer (MCP). The MCP bonding mechanism likely involves the MMA monomer penetrating the resin matrix of the CB followed by polymerization [15,16]. These two primers have completely different bonding mechanisms and numerous studies have reported their bonding performances to CBs [15,16,17,18,19,20,21,22,23,24,25,26,27,28].

CBs possess a broad range of microstructures that play a significant role in their bonding properties [29]. Previous studies have reported that the bonding properties of CBs are dependent on the material used. However, further evaluation is required to assess the bonding properties of various types of CBs [15,24,25,27]. Additionally, it is important to evaluate the bonding properties not only on sandblasted but also on non-sandblasted surfaces. Although sandblasting has been reported to enhance bonding strength and is currently an essential pretreatment for bonding CBs in clinical practice [30], adequate sandblasting of the entire inner surface of restorations is difficult. It is advisable to minimize sandblasting, especially in proximity to the margins, as it has the potential to increase the marginal gap and create a rough outer surface near the margin, which can contribute to greater biofilm formation [31]. The interface between a cemented restoration and tooth is susceptible to recurrent caries due to luting agent dissolution and inherent interface roughness [32]. Previous research has indicated that better marginal fit [33,34,35] and reduced surface roughness near the margin [33] are associated with lower risks of periodontal disease. Considering these findings, it is plausible to suggest that insufficient sandblasting near the margins could be a contributing factor to the debonding of CAD/CAM composite crowns. Consequently, the bond strength of non-sandblasted surfaces is deemed a crucial factor. It is important to note that, to the best of our knowledge, no previous studies have specifically explored this perspective, and the available information, particularly regarding MCP treatment, is limited. Only one study has assessed the bond strength for MCP treatments on non-sandblasted surfaces [16].

The purpose of this study is to evaluate the bonding characteristics of the two types of primer. To achieve this, shear bond strengths (SBSs) of bonding systems using SCA and MCP were measured for 11 types of CBs with and without sandblasting. The null hypothesis of this study is that there are no differences in bond strength among CBs under the same bonding conditions.

## 2. Materials and Methods

### 2.1. Materials

In this study, two types of bonding systems—a silane coupling agent (Ceramic Primer II, GC Corp., Tokyo, Japan) and an MMA-containing primer (Block HC Cem, Shofu, Kyoto, Japan)—were investigated. Eleven commercial CBs for the CAD/CAM system were chosen: Cerasmart 270 (C270), Cerasmart 300 (C300), Shofu block HC (HC), Shofu block HC hard (HCh), Katana Avencia block (Ka), Katana Avencia P block (KaP), Estelite block (Est), Estelite P block (EstP), KZR-CAD HR2 (KZ2), KZR-CAD HR3 GAMMATHETA (KZ3), VITA Enamic (Ena). Detailed properties of the bonding systems and the CBs are listed in Table 1 and Table 2, respectively.

### 2.2. Shear Bond Strength Testing

The CBs were cut into plates with a thickness of 2.5 mm using a precision sectioning diamond saw (Isomet LS, Buehler, Lake Bluff, IL, USA). The plates were then embedded in cold-setting epoxy resin (Epofix, Struers, Copenhagen, Denmark). The bonding surfaces were automatically ground under water irrigation using a polishing machine (Ecomet III /Automet II, Buehler, Lake Bluff, IL, USA) with a series of silicon carbide abrasive papers up to P600.

Thirty-two specimens of CBs were prepared and divided into two groups (*n* = 16). The first group, called the sandblasted group, was subjected to sandblasting using alumina particles having a grain size of 50 μm (Alumina polishing material, Sintokogio, Ltd., Nagoya, Japan), administered to the bonding area in small circular movements. The standoff distance was 15 mm, with a pressure of 0.2 MPa for 2 s. The other group did not receive any treatment, and was called the non-sandblasted group. Specimens were subsequently cleaned ultrasonically with distilled water for 5 min. Both groups were further divided into SCA and MCP subgroups (*n* = 8). For the SCA groups, Ceramic Primer II (GC Corp., Tokyo, Japan) was applied onto the surface and dried with oil-free air. For the MCP groups, a layer of HC primer (Shofu Inc., Kyoto, Japan) was coated onto the surface, as thinly as possible, by blowing with oil-free air. The HC primer was then light-cured for 15 s using a light-emitting diode (LED) light-curing unit (VALO V25460, Ultradent Products Inc., South Jordan, UT, USA) with an output of 1000 mW/cm^2^. A polypropylene mold with an opening having a diameter of 2.38 mm and height of 2.0 mm (Ultradent Products Inc., South Jordan, UT, USA) was positioned on the bonding area of the specimens. The resin cements were mixed and packed into the mold and then light-cured for 30 s using the LED light-curing unit. After extraction from the molds, all bonded specimens were stored in distilled water for 24 h at 37 °C.

Notched-edge SBS tests were conducted according to ISO 29022:2013 [36]. The bonded specimens were loaded at a crosshead speed of 1.0 mm/min using a mechanical testing machine (Model 4481, Instron Corporation, Canton, MA, USA). After the testing, the failure mode was determined using an optical microscope (KH-7700, Hirox Co., Tokyo, Japan,) and classified into three categories: (a) adhesive failure between CB and resin cement (which was defined in this study as more than 90% of failure at the CB-cement interface); (b) mixed failure; and (c) cohesive failure within CB.

### 2.3. Surface Roughness Measurement

The arithmetic mean roughness (Ra) of non-sandblasted and sandblasted surfaces of each CB (*n* = 5) was measured using a tactile roughness measuring device (Surfcom 130A, Accretech, Tokyo, Japan) with a threshold of 0.8 mm as the cut-off value, a measurement length of 5.0 mm, a measurement speed of 0.6 mm/s, and by applying a Gaussian filter. The specimens were prepared in a manner similar to that of the SBS tests. Additionally, an as-milled surface produced by the CAD/CAM machine (Ceramill Motion 2, Amann Girrbach AG, Herrschaftswiesen, Austria) was measured for C270, which was chosen as the representative of the CBs. The specimens were prepared by machining into a cube with a length of 10 mm (*n* = 5).

### 2.4. Surface Observation

Non-sandblasted and sandblasted surfaces of each CB were prepared by the same procedure as that for the SBS test and were then observed. To characterize the CB microstructure, mirror-polished surfaces of the CBs were prepared by polishing with a sequence of abrasives down to a 0.3 μm alumina suspension using a polishing machine (Ecomet III, Buehler, Lake Bluff, IL, USA). Secondary electron images were obtained using an electron probe microanalyzer (JXA-8530FA, JEOL, Tokyo, Japan) at an accelerating voltage of 10 kV after platinum coating.

### 2.5. Statistical Analysis

Before analysis, homogeneity of variance (Bartlett’s test) and normal distribution (Shapiro–Wilk test) were confirmed for the data. The SBS data were analyzed using Student’s *t*-test to identify variations in SBS between primer types for each CB. Furthermore, a two-way ANOVA was employed to examine the interplay between sandblasting and primer type for each CB. Additionally, Tukey’s multiple comparison test was conducted to discern disparities in SBS and Ra across the various CBs. Statistical analysis was performed using SPSS software (Version 27.0, SPSS Inc., Chicago, IL, USA). *p* values smaller than 0.05 were considered statistically significant in all tests. To check the appropriateness of the sample size, a post hoc power analysis was performed (G*Power 3.1.9.7, Heinrich Heine University, Düsseldorf, Germany).

## 3. Results

### 3.1. Power Analysis

A sample size of eight and five per group was used, providing 80% power to detect effect sizes of 0.45 and 0.60, respectively, when conducting a single-factor ANOVA at the alpha level of 0.05. For *t*-tests and two-way ANOVA, a sample size of eight per group yielded 80% power to detect effect sizes of 1.50 and 0.51, respectively.

### 3.2. Shear Bond Strength Testing

The SBS results are shown in Table 3. Statistically significant differences were observed between CBs within all identical conditions. In the non-sandblasted groups, the specimens treated with MCP showed significantly higher SBSs than those with SCA for all CBs except KZR3 and Ena; all non-sandblasted specimens exhibited adhesive failure. In the sandblasted groups, the MCP-treated specimens showed significantly higher SBSs than the SCA-treated specimens in seven of the 11 types of CBs, while the reverse was true for Est, KZR3, and Ena.

**Figure 1:**
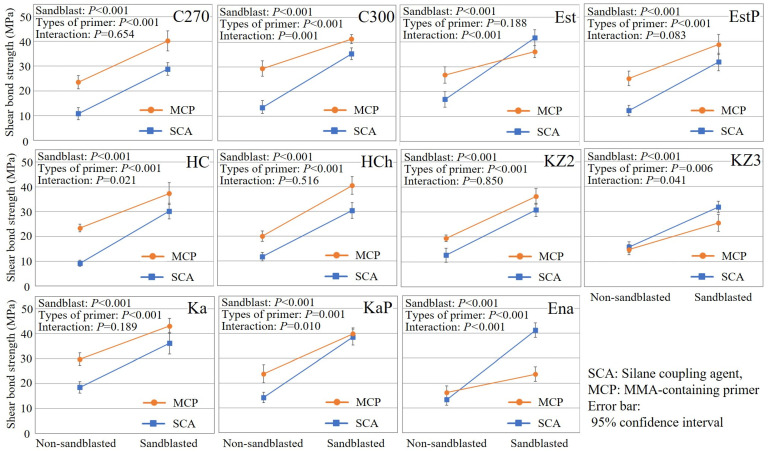
Analysis of two-way ANOVA for shear bond strength (SBS) results.

The results of two-way ANOVA for SBS are shown in Figure 1. The interaction between sandblasting and the types of primer was statistically significant for C300, HC, KaP, Est, KZ3, and Ena. Especially for Est and Ena, the sandblasting treatment significantly improved the SBS of the SCA group, and the crossover effect was observed.

### 3.3. Surface Roughness Measurement

The results of surface roughness measurements of CBs are listed in Table 4. No statistically significant differences in Ra were observed among the CBs in the non-sandblasted groups, nor between the as-milled by CAD/CAM machine surface and the non-sandblasted surfaces of all CBs. In the sandblasted groups, although HC showed a statistically significant higher Ra than that of some CBs (Est, EstP, KZ2, KZ3, Ena), no statistically significant differences were observed among all CBs except HC.

### 3.4. Surface Observation

**Figure 2:**
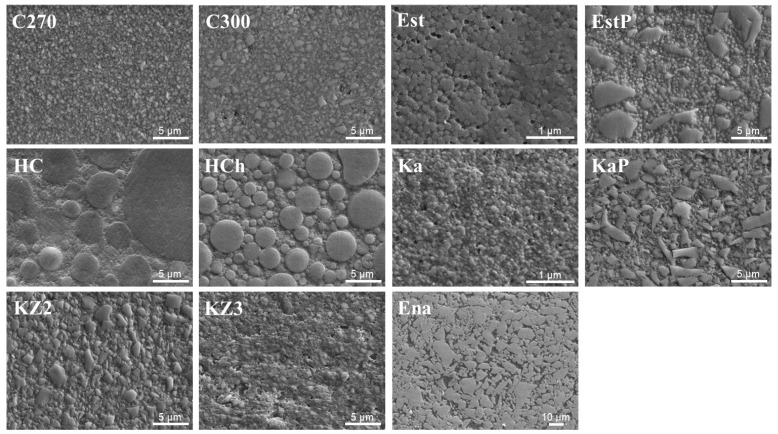
Secondary electron images (5000× original magnification except for Est, Ka, Ena) of polished surfaces for each CB. Original magnification for Est and Ka is 30,000×, 1000× for Ena.

**Figure 3:**
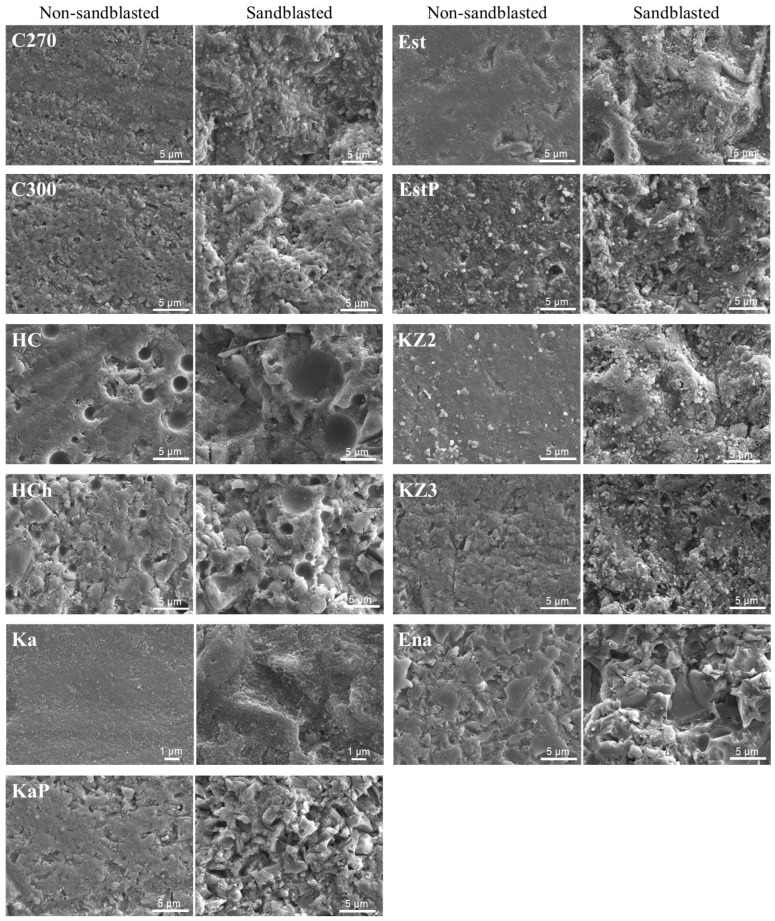
Secondary electron images (5000× original magnification except for Ka) of both non- and sandblasted surfaces for each CB. Original magnification for Ka is ×10,000.

The polished surface observations (Figure 2) revealed that the microstructures of the fillers varied widely among the CBs. In the non-sandblasted groups (Figure 3), which were ground using abrasive papers (P600), the exposure of fillers was not clear, while in sandblasted groups, exposed fillers were clearly observed, and the surface appearances differed significantly among the CBs.

## 4. Discussion

CBs have a varied composition, particularly in terms of filler contents, particle size, and distribution; thus, their bonding properties are expected to be material dependent. The results of this study demonstrated that the CB microstructures varied widely based on the surface observations, and that there were statistically significant differences in SBS between the CBs within all groups. Therefore, the null hypothesis in this study was rejected. In addition, surface roughness analysis indicated that there was no statistically significant disparity in Ra between CBs (except for HC) in both the non-sandblasted and sandblasted groups. This suggests that any variations in SBS between CBs within each group cannot be attributed to differences in surface roughness.

In a clinical setting, the surface of CBs prior to sandblasting is the one produced by CAD/CAM milling. In this study, the surface in the non-sandblasted groups was ground using abrasive papers (P600) to be representative of the as-milled surface [17]. This assumption was supported by the results where no significant differences in surface roughness (Ra) were observed between the as-milled by the CAD/CAM machine surface and the surfaces ground by the abrasive papers (P600).

For the non-sandblasted groups, the specimens treated with SCA showed significantly lower bond strength, consistent with findings from other studies [16,17]. In nine out of the 11 types of CBs, the MCP-treated specimens showed a significantly higher SBS than those in the SCA-treated group. For some CBs, the SBS of the former was more than twice as high as that of the latter group. This trend corroborates data from a previous study that reported that non-sandblasted specimens treated with MCP had higher bond strength than those treated with SCA in all four types of CBs investigated [16]. These results can be attributed to the difference in bonding properties between the two primers, with MCP and SCA requiring adhesion to the resin matrix and fillers, respectively. In fact, the surface observations of the non-sandblasted specimens revealed the presence of more resin matrix rather than exposed fillers. Based on these findings, it can be inferred that MCP exhibits more favorable bonding characteristics compared to SCA, particularly in proximity to the margins. This observation aligns with the clinical perspective advocating for the minimization of sandblasting near the margins, as highlighted in the introduction. Conversely, for the other two CBs, KZ3 and Ena, there were no statistically significant differences in SBS between the SCA and MCP groups. Although this might be due to differences in matrix resin composition or a greater number of exposed fillers on the surfaces than in the other CBs, no clear explanation was found in this study owing to limited information.

In this study, sandblasting of the bonding area was performed for a duration of 2 s. During the preliminary experiment, it was observed that longer sandblasting led to surface depressions that were visually evident on some materials. These depressions increased the distance between the bonding surface and the loading point in the SBS test, potentially resulting in lower measured bond strength than the actual bond strength. To mitigate this effect, a sandblasting time of 2 s was chosen to avoid the formation of such depressions and ensure more accurate bond strength measurements. In the sandblasted groups, statistically significant differences in SBS were observed only for KZ3 and Ena in the MCP group; in the SCA group, significant differences were confirmed among CBs, and their SBSs were more material dependent. The latter result could be attributed to the fact that the effectiveness of SCA relies on the exposure of filler particles on the surface, and thus, the bond strength is greatly affected by the various microstructures of the CBs. For HC, EstP, KZ2, and KZ3, the sandblasted SCA-treated specimens exhibited low SBS, while adhesive failure was observed in all specimens. Observation of the sandblasted surfaces of EstP, KZ2, and KZ3 revealed an abundant number of fillers that were not integrated with the resin matrix, but were only adhered to the surface. Although more evidence is needed, the low SBS results are expected to be caused by the presence of these fillers. Furthermore, numerous voids due to filler desorption, but not exposed fillers, were observed on the sandblasted surface of HC [17,28], which may account for the low SBS for HC. Comparing SBS between the SCA and MCP groups, seven out of 11 CBs showed significantly higher SBSs for the MCP group. This trend is in accordance with the results of other studies [15,16,24,26]. Although the MCP treatment showed superior bonding properties, it should be noted that MCP treatment did not consistently offer a strong bond for all CBs. In fact, KZ3 and Ena exhibited low SBSs around 25 MPa with adhesive failure between the CB and the resin cement in all specimens. Another study involving Ena also reported that MCP-treated specimens exhibited lower bond strength compared to SCA-treated specimens [25]. Ena is classified as PICN, which has a high filler content of 86 wt.%. The low SBS of Ena can be attributed to its high filler content, which is the highest among all the CBs. On the other hand, the reasons behind the low SBS of KZ3 remain unclear. Given that no significant differences in the surface roughness were observed between KZ3 and all CBs except for HC and that no specific surface features were noted from the surface observation, factors other than surface properties may be involved.

The results of two-way ANOVA for SBS demonstrated that the interactions between sandblasting and the types of primer were statistically significant for some CBs and that sandblasting was more effective for the SCA group than for the MCP group. Additionally, especially for Est and Ena, the sandblasting treatment significantly improved the SBS of specimens treated with SCA, and a crossover effect was observed. These results clearly suggest that sandblasting not only improves bond strength through both micro-mechanical retention and increased surface area, but also enhances the effectiveness of SCA by exposing filler surfaces. While this study lacks direct evidence, the findings obtained do align with the notion that the elevated rate of debonding of CAD/CAM composite crowns could be attributed to inadequate sandblasting near the margins, particularly in cases where SCA is employed.

The observations of the failure surfaces showed some cohesive failures within the CBs for the sandblasted specimens, in which the surface around the bonding area was deeply hollowed out; similar fracture surfaces have been reported in other studies [19,22]. Cohesive failure within the CB implies that the bond strength between CB and resin cement exceeded the fracture resistance of the CB itself, and thus, these results suggest that excessive stress concentration may have occurred in the CB in the shear bond strength test. Moreover, Yoshihara et al. reported that sandblasting damaged surfaces of CBs with cracks of 1–10 μm, and for a certain CB (HC), it caused serious damage and adversely affected the bond strength [28]. These findings suggest that CB surfaces were damaged by sandblasting, thereby causing cohesive failure within the CBs. Therefore, it is possible that the SBS results in samples with cohesive failures were dependent on fracture resistance under the stress concentration of the CBs damaged by sandblasting, which is one limitation of this study. In fact, SCA-treated specimens for C270 and HCh showed low SBS values of approximately 30 MPa, even though certain specimens exhibited cohesive failure within the material and no specimens exhibited adhesive failure. However, MCP-treated specimens for C270 and HCh showed higher SBS values of approximately 40 MPa while exhibiting similar fracture modes as the SCA-treated specimens. Hagino et al. reported that MCP migrates into the microcracks induced by sandblasting and can subsequently cure in those regions [24]. Thus, the above findings can be explained by the MCP treatment filling the microcracks with cured MCP and reducing the damage to the surface caused by sandblasting.

Another limitation of this study is that the influence of material aging on bond strengths was not evaluated. A previous study reported that MCP provided greater adhesion to CBs even over prolonged storage compared with an SCA treatment, and that the use of the former primer is recommended when bonding CAD/CAM composite restorations [24]. However, the evaluation of various CBs after an aging period is incomplete and needed for further studies.

## 5. Conclusions

The primary conclusions of this study are as follows:

1. Although the MCP treatment did not consistently achieve a strong bond for all CBs, it was more effective than the SCA treatment, especially for non-sandblasted surfaces.
2. The effect of the SCA treatment was more material-dependent than the MCP treatment.
3. Sandblasting is an important process for achieving a strong bond, and particularly essential for the SCA treatment.

## Figures and Tables

**Table 1:** Bonding systems used in this study.

Bonding System	Code	Manufacture	Product (Lot No.)	Composition *
Bonding system using silane coupling agent	SCA	GC Corp., Tokyo, Japan	Resin cement	phosphoric acid ester monomer, methacrylic acid ester, fluoro alumino silicate glass, silica filler
			G-CEM (1810041)	
			Primer	vinyl silane, methacrylic acid ester, ethanol
			Ceramic Primer II (1901091)	
Bonding system using MMA-containing primer	MCP	Shofu Inc., Kyoto, Japan	Resin cement	fluoro alumino silicate glass, glass powder, UDMA, 2-HEMA, carboxylic acid-based monomer, phosphonic acid-based monomer, zirconium silicate, reaction initiator
			Block HC Cem (081819)	
			Primer	UDA, MMA, acetone, polymerization initiator
			HC Primer (081819)	

MMA: methyl methacrylate, UDMA: urethane dimethacrylate, HEMA: hydroxyethyl methacrylate. * Data provided by manufacturer.

**Table 2:** Composite blocks (CBs) used in this study.

Brand	Code	Manufacturer	Lot No.	Composition *		
				Monomer	Filler	
					Composition	Content (wt%)
Cerasmart 270	C270	GC Corp., Tokyo, Japan	1708223	UDMA	zirconia-silica filler barium glass	77
Cerasmart 300	C300	GC Corp.	1712112	UDMA	zirconia-silica filler barium glass	78
Shofu block HC	HC	Shofu Inc., Kyoto, Japan	121501	UDMA, TEGDMA	silica powder, micro fumed silica, zirconium silicate	62
Shofu block HC hard	HCh	Shofu Inc.	817667	UDMA, TEGDMA	zirconium silicate micro fumed silica	69.5
Katana Avencia block	Ka	Kuraray Noritake Dental Inc., Tokyo, Japan	000162	UDMA, TEGDMA	alumina, silica filler	62
Katana Avencia P block	KaP	Kuraray Noritake Dental Inc.	000024	UDMA, TEGDMA	barium glass, silica filler	82
Estelite block	Est	Tokuyama Dental Corp., Tokyo, Japan	032068	Bis-MPEPP, UDMA, NPGDMA	silica powder, silica-zirconia filler	75
Estelite P block	EstP	Tokuyama Dental Corp.	003048	Bis-MPEPP, UDMA, NPGDMA	silica powder, silica-zirconia filler	81
KZR-CAD HR2	KZ2	Yamakin Co., Ltd., Osaka, Japan	01121522	UDMA, DEGDMA	SiO_2_-Al_2_O_3_-ZrO_2_, SiO_2_	72
KZR-CAD HR3 GAMMATHETA	KZ3	Yamakin Co., Ltd.	02011812	UDMA, DEGDMA	SiO_2_-Al_2_O_3_-ZrO_2_, SiO_2_	75
VITA Enamic	Ena	Vita Zahnfabrik, Bad Sackingen, Germany	51530	UDMA, TEGDMA	fine-structure feldspar ceramic enriched with aluminum oxide (Polymer Infiltrated Ceramic Network)	86

UDMA: urethane dimethacrylate; Bis-MPEPP: 2,2-Bis (4-methacryloxypolyethoxyphenyl) propane; NPGDMA: neopentyl glycol dimethacrylate; DEGDMA: diethylene glycol dimethacrylate; TEGDMA: triethylene glycol dimethacrylate. * Data provided by manufacturer.

**Table 3:** Results of shear bond strength (SBS) test.

Composite Block (CB)	Non-Sandblasted Group					Sandblasted Group				
	Treatment					Treatment				
	Silane Coupling Agent		MMA-Containing Primer		*p*-Value (*t*-Test)	Silane Coupling Agent		MMA-Containing Primer		*p*-Value (*t*-Test)
	Shear Bond Strength	Fracture Mode	Shear Bond Strength	Fracture Mode		Shear Bond Strength	Fracture Mode	Shear Bond Strength	Fracture Mode	
C270	10.8 (3.3) ^cd^	[8/0/0]	23.5(3.6) ^abc^	[8/0/0]	<0.001	28.8 (3.5) ^c^	[0/5/3]	40.2 (5.4) ^a^	[0/5/3]	<0.001
C300	13.5(3.8) ^abcd^	[8/0/0]	29.3 (4.3) ^ab^	[8/0/0]	<0.001	35.3 (3.2) ^abc^	[3/5/0]	41.3 (2.4) ^a^	[0/5/3]	0.001
HC	9.2 (1.6) ^d^	[8/0/0]	23.4 (2.2) ^bc^	[8/0/0]	<0.001	30.2 (4.2) ^c^	[8/0/0]	37.3 (5.9) ^a^	[0/4/4]	0.022
HCh	11.7 (2.4) ^bcd^	[8/0/0]	20.0 (2.9) ^cd^	[8/0/0]	<0.001	30.4 (4.3) ^c^	[0/5/3]	40.5 (4.9) ^a^	[0/3/5]	0.001
Ka	18.5 (3.1) ^a^	[8/0/0]	29.8 (3.5) ^a^	[8/0/0]	<0.001	36.2 (5.8) ^abc^	[0/5/3]	43.1 (4.2) ^a^	[0/3/5]	0.023
KaP	14.2(2.8) ^abcd^	[8/0/0]	23.7(4.9) ^abc^	[8/0/0]	0.001	38.5 (4.4) ^ab^	[0/3/5]	39.8 (3.2) ^a^	[5/3/0]	0.523
Est	16.9 (4.3) ^ab^	[8/0/0]	26.7 (4.5) ^ab^	[8/0/0]	0.001	41.7 (4.4) ^a^	[0/5/3]	36.1 (3.3) ^a^	[0/1/7]	0.017
EstP	12.3 (2.8) ^bcd^	[8/0/0]	25.1(3.9) ^abc^	[8/0/0]	<0.001	31.7 (4.7) ^bc^	[8/0/0]	38.7 (5.5) ^a^	[0/6/2]	0.022
KZ2	12.7(2.4) ^abcd^	[8/0/0]	19.4 (1.8) ^cd^	[8/0/0]	0.002	30.8 (3.6) ^c^	[8/0/0]	36.2 (4.2) ^a^	[0/7/1]	0.020
KZ3	15.8 (2.8) ^abc^	[8/0/0]	14.8 (2.8) ^d^	[8/0/0]	0.507	31.8 (3.0) ^bc^	[8/0/0]	25.4 (4.5) ^b^	[8/0/0]	0.007
Ena	13.4(3.2) ^abcd^	[8/0/0]	16.2 (3.6) ^d^	[8/0/0]	0.150	41.2 (4.0) ^a^	[0/0/8]	23.6 (3.9) ^b^	[8/0/0]	<0.001

Unit: MPa, *n* = 8, values in parentheses indicate standard deviations. [The number of specimens in each fracture mode]: [adhesive failure/mixed failure/cohesive failure within CB]. Same lowercase letters in a same column indicate no significant differences (*p* > 0.05).

**Table 4:** Surface roughness (Ra) of CBs.

CB	Group	
	Non-Sandblasted	Sandblasted
C270	0.189 (0.044) ^a^	0.922 (0.076) ^ab^
C300	0.172 (0.016) ^a^	0.973 (0.114) ^ab^
HC	0.182 (0.017) ^a^	1.107 (0.176) ^b^
HCh	0.197 (0.030) ^a^	0.986 (0.139) ^ab^
Kat	0.188 (0.033) ^a^	0.909 (0.087) ^ab^
KatP	0.212 (0.037) ^a^	0.918 (0.067) ^ab^
Est	0.186 (0.020) ^a^	0.856 (0.080) ^a^
EstP	0.189 (0.019) ^a^	0.850 (0.111) ^a^
KZ2	0.201 (0.028) ^a^	0.860 (0.087) ^a^
KZ3	0.189 (0.042) ^a^	0.824 (0.028) ^a^
Ena	0.198 (0.020) ^a^	0.835 (0.046) ^a^
	As-milled surface by CAD/CAM machine	
C270 *	0.188 (0.017) ^a^	-

Unit: µm. Same lowercase letters in a same column indicate no significant differences (*p* > 0.05). * C270 was chosen as a representative of the CBs.

## Data Availability

The data presented in this study are available on request from the corresponding author. The data are not publicly available due to its confidentiality.
